# Impact of copper and zinc oral chronic exposure on Carniolan honey bee survival and feeding preference

**DOI:** 10.1093/jee/toae108

**Published:** 2024-05-15

**Authors:** Gordana Glavan, Grega Benko, Janko Božič

**Affiliations:** Department of Biology, Biotechnical Faculty, University of Ljubljana, Jamnikarjeva 101, SI-1000, Ljubljana, Slovenia; Department of Biology, Biotechnical Faculty, University of Ljubljana, Jamnikarjeva 101, SI-1000, Ljubljana, Slovenia; Department of Biology, Biotechnical Faculty, University of Ljubljana, Jamnikarjeva 101, SI-1000, Ljubljana, Slovenia

**Keywords:** CuSO_4_, ZnCl_2_, LC_50_, pollinator, pollution

## Abstract

Honey bees are important plant pollinators and honey producers. Contamination of the environment with metals can lead to a decline in honey bee populations. Copper (Cu) and zinc (Zn) salts are commonly used as fungicides and foliar fertilizers. In this study, we investigated the effects of 10-day chronic oral exposure to different concentrations of Cu (CuSO_4_) and Zn (ZnCl_2_) on survival and feeding rates of Carniolan honey bees in laboratory conditions. We found that mortality in honey bee workers increased in a concentration-dependent manner and that Cu (lethal concentration [LC_50_] = 66 mg/l) was more toxic than Zn (LC_50_ = 144 mg/l). There was no difference in the feeding rate of Cu-treated bees for the different concentrations tested, but the feeding rate decreased with the increase in Zn concentration. To determine feeding preference or avoidance for Cu and Zn, we conducted 2-choice 24-h feeding experiments. We demonstrated that honey bees preferred Zn-containing solutions compared to the control diet. A two-choice experiment with Cu showed a tendency for honey bees to be deterred by Cu at high concentrations; however, it was not statistically significant. In summary, our results suggest that honey bee workers may suffer adverse effects when exposed to ecologically relevant concentrations of Cu and Zn.

## Introduction

Environmental contamination with pesticides and other xenobiotics, such as metals, can result in a reduction of honey bee (Apidae: Hymenoptera) populations (vanEngelsdorp and Meixner 2010). Anthropogenic emissions, especially industry, are the main cause of environmental contamination with metals. Metals can be found in air, soil, and water (Lloyd and Showak 1984, Schacklette and Boerngen 1984, Holmgren et al. 1993, Meng et al. 2016). Major routes of honey bee exposure are nectar and pollen from plants that have been contaminated through the absorption from soil and by a deposition of atmospheric particles on flowers due to topsoil erosion (Nagajyoti et al. 2010). Metals can easily be adsorbed by honey bee cuticula and accumulate in different honey bee tissues and royal jelly (Leita et al. 1996, Fakhimzadeh and Lodenius 2000, Roman 2010, Giglio et al. 2017). Subsequently, metals are found in most honey bee products and thus have an impact on not just foragers, but the whole colony (Roman 2010, Roman et al. 2011). The exposure duration of honey bees to metals in contaminated areas is usually prolonged due to metal accumulation in the environment and frequent visits of these areas by foragers. Sublethal chronic impact of metals on honey bees is common in contaminated areas, negatively influencing their fitness and cognitive abilities and thus impairing the viability of the colonies (Johnson et al. 2010, Hladun et al. 2012, Søvik et al. 2015, Burden et al. 2016). Negative acute and chronic effects of metals, especially heavy metals, such as Cu, Zn, Cd, and Pb, have been reported, but the number of studies is limited. Chronic and acute toxicity tests with Cd, Cu, and Pb on development and survival of honey bee were evaluated by Di et al. (2016). Joint effects of Cd and Cu in 1:1 concentration ratio demonstrated increased larval developmental duration, the mortality of both larvae and foragers, decreased forager feeding, and altered the sucrose response behavior of foragers (Di et al. 2020). Selenium (Se) can also have negative effect on honey bee behavior and survival, by disrupting associative conditioning and long-term memory recall (Hladun et al. 2012, Burden et al. 2016), whereas Mn had a negative impact on foraging (Søvik et al. 2015). Hladun et al. (2016) treated honey bee colonies with Cd, Se, Pb, or Cu and found Cu to be the least toxic to larvae with an LC_50_ of 6.97 mg/l and Pb for foragers with an LC_50_ of 345 mg/l. Comparing the same exposure concentrations, Se-treated colonies had lower total worker weights, while Pb showed a minimal effect. Bounias et al. (1996) showed sublethal detrimental effects of cupric salts gluconate, sulfate influence gut alkaline, and acid phosphatases in honey bees. The exposure to metal oxide nanoparticles, such as Ag-TiO_2_, ZnO-TiO_2_, and TiO_2_ (Özkan et al. 2015), ZnO (Milivojević et al. 2015), cerium (IV) oxide (CeO_2_) (Kos et al. 2017), PbO, and CdO (Naggar et al. 2014) negatively affects honey bee survival, feeding, detoxifying enzymes, and neuronal system.

Mining, smelting metals, and steel production, as well as burning coal and certain wastes can release zinc into the environment (Meylan and Reck 2017). Anthropogenic sources of diffuse Cu contamination include fungicidal treatments, liquid manure (mainly from pigs), sewage sludge, atmospheric deposition, mining activities, local industrial contamination, and particles from car brakes (Panagos et al. 2018). Cu and zinc Zn salts are often used as fungicides or leaf fertilizers. In nonindustrial environment, the most likely routes of honey bee intoxication are probably direct pesticide overspray, water droplets, and by plant guttation of water, pollen, and nectar (Johnson 2015). Honey bees can also be in contact with Zn and Cu also in hives containing zinc-plated steel parts and wooden components treated with preservatives (Kalnins and Detroy 1984, Milivojević et al. 2015). However, the optimal dietary Zn levels could be beneficial to honey bees by increasing their antioxidant defenses or even defending against heavy metals toxicity (Zhang et al. 2015, Nisbet et al. 2018).

Given the presence of Cu and Zn in the environment, accumulation in honey bees and honey bee products, their frequent use as fungicides or fertilizers, and the lack of studies on the sublethal effects on honey bees, we aimed to evaluate the effects of chronic exposure of different concentrations of Cu and Zn on honey bee survival and feeding rates. To assess feeding preference or avoidance for Cu and Zn, we performed 2-choice feeding experiments, where bees were able to choose between the control sucrose diet and solutions with different Cu or Zn concentrations. We presume that honey bees accumulate metals in their body most intensively *via* contaminated food such as nectar, thus we exposed them orally to Zn and Cu in sucrose solution. The feeding preference test was used to find out if honey bees prefer contaminated over unpolluted food. This preference could lead to higher consumption and thus potentiate the intoxication of bees by Cu or Zn.

## Materials and Methods

Carniolan adult worker honey bees *Apis mellifera carnica*, Pollman 1879 (Insecta, Hymenoptera: Apidae) were employed in all experiments. Honey bee colonies were maintained according to good beekeeping standard (Kulhanek et al. 2021) at the Department of Biology, Biotechnical Faculty, University of Ljubljana, Ljubljana, Slovenia (coordinates 46.051898, 14.468446). They were not treated with any chemical substances at least 1 month before the onset of the experiments. Only summer adult worker honey bees were used, and they were collected from June to August from brood combs inside the hive. Healthy undamaged honey bee workers were transferred directly into the cages through the cage openings with an aspirator without anesthesia. Water and 1.5 M sucrose solution were offered to the bees during the collection. After collection, the caged bees were transferred to an incubator (34 °C, 60% RH) and provided with dechlorinated water 2 h before the onset of feeding exposures. All tested solutions were administered to bees by graduated sterile syringes as described in detail by Glavan (2020). In the following experiments, Cu was applied as CuSO_4_, and Zn as ZnCl_2_. The CuSO_4_ (Sigma-Aldrich Co., Germany) and ZnCl_2_ (Sigma-Aldrich Co., Germany) solutions were prepared in a 1.5 M sucrose (Sigma-Aldrich Co., Germany) (51.35% w/v) solution. All experiments were conducted in an incubator (34 °C, 60% RH).

In the first set of experiments, the effects of 10-day oral feeding exposure of bees to sublethal concentrations of Cu and Zn were tested. The exposure concentrations range and time of exposure were chosen based on preliminary testing (data not shown) and previous studies (Milivojević et al. 2015, Glavan et al. 2017). For both Cu and Zn chronic 10-day experiments, honey bees were distributed to test cages (10 × 6 × 7 cm; length × width × height), made of wood, steel wire mesh, and sliding transparent glass, as described in detail previously (Glavan et al. 2018). Each test cage was supplied ad libitum with 2 feeders, one containing 1.5 M sucrose, with or without testing substance, and another containing dechlorinated water. The feeders were gravity feeders with graduated sterile syringes for single use with cut open ends (polypropylene + polyethylene; Ecoject, Dispomed, Germany). All liquids were renewed every 24 h during the exposure period. In both chronic experiments, with Cu or Zn, 3 cages of bees were treated with each exposure concentration of tested substance (total number of cages = 15 per experiment; 10–15 bees per cage). The exposure Cu concentrations and total number of bees per treatment were: 0 mg/l (*N* = 33), 15.9 mg/l (*N* = 32), 31.8 mg/l (*N* = 33), 63.6 mg/l (*N* = 33), and 127.1 mg/l (*N* = 34). The exposure Zn concentrations and a total number of bees per treatment were: 0 mg/l (*N* = 38), 32.7 mg/l (*N* = 42), 65.4 mg/l (*N* = 39), 130.8 mg/l (*N* = 42), and 261.6 mg/l (*N* = 44). The mortality and amount of consumed food was recorded by weighing the gravity feeders every 24 h. Both chronic Cu and Zn experiments were run for 10 days.

In the second set of experiments, two 2-choice feeding experiments were performed, one with Cu, and the second with Zn. With these experiments, we aimed to explore how honey bees distribute their feeding between 2 qualities of food to find out if they develop any deterrence or preference for tested metals. Each group of honey bees could choose between a 1.5 M of sucrose solution with different concentrations of Cu or Zn and a 1.5 M of sucrose solution. For both 2-choice experiments, honey bees were distributed to plastic hoarding cages (9 × 7 × 7 cm; length × width × height) containing a removable top, multiple ventilation holes and 3 holes for gravity feeders (for reference, see Williams et al. 2013). The feeders were placed at the top of the cages and were extended almost to the bottom. The gravity feeders had the same characteristics as in the first set of experiments. Other than the 2 test feeders, another feeder was filled with dechlorinated water. In both 2-choice experiments, 3 cages of bees were used per each concentration of tested substance (total number of cages = 15 per experiment; 15–20 bees per cage). In the first Cu 2-choice feeding experiment, one gravity feeder with the control 1.5 M sucrose solution was applied to each cage. In addition, each cage was supplied with a second gravity feeder with a test solution: a 1.5 M sucrose solution containing Cu with one of five different concentrations. The exposure Cu concentrations and a total number of bees per concentration were: 0 mg/l (*N* = 50), 15.9 mg/l (*N* = 51), 31.8 mg/l (*N* = 48), 63.6 mg/l (*N* = 46), and 127.1 mg/l (*N* = 51). For the Zn 2-choice feeding experiment, the methodology was the same: one gravity feeder with the control 1.5 M sucrose solution was applied to each cage. Each cage was also supplied with a second gravity feeder with a test solution: a 1.5 M sucrose solution containing Zn with 1 of 5 different concentrations. The exposure Zn concentrations and total number of bees per concentration were: 0 mg/l (*N* = 57), 32.7 mg/l (*N* = 52), 65.4 mg/l (*N* = 54), 130.8 mg/l (*N* = 54), and 261.6 mg/l (*N* = 55). The weight of consumed food was recorded by weighing the gravity feeders at the end of experiments. Both 2-choice Cu and Zn experiments were run for 24 h.

The differences in survival of worker bees during the chronic studies were tested using Kaplan–Meier survival statistics with the log-rank test (Mantel–Haenszel test; Kaplan and Meier 1958). The feeding rate was calculated as the amount of food eaten per number of live honey bees in the cage per day. The differences in feeding rate in chronic experiments were tested with Kruskal–Wallis test and Dunn’s post hoc test (Kruskal and Wallis 1952, Dunn 1964). The consumption of food in 2-choice experiments was tested using Wilcoxon test (for paired samples) and GLM procedure (Wilcoxon 1945). R Programming environment (http://www.rproject.org/) and R-studio were used for all statistical analyses described above except for Kaplan–Meier survival statistics that were carried out using the program GraphPad Prism (GraphPad Software, USA). Medial lethal concentration (LC_50_) for 10-day oral treatments was calculated by use of Probit analysis calculator (https://probitanalysis.wordpress.com/2016/07/07/first-blog-post/) based on the method of Finney (1952).

## Results

**Fig. 1.**
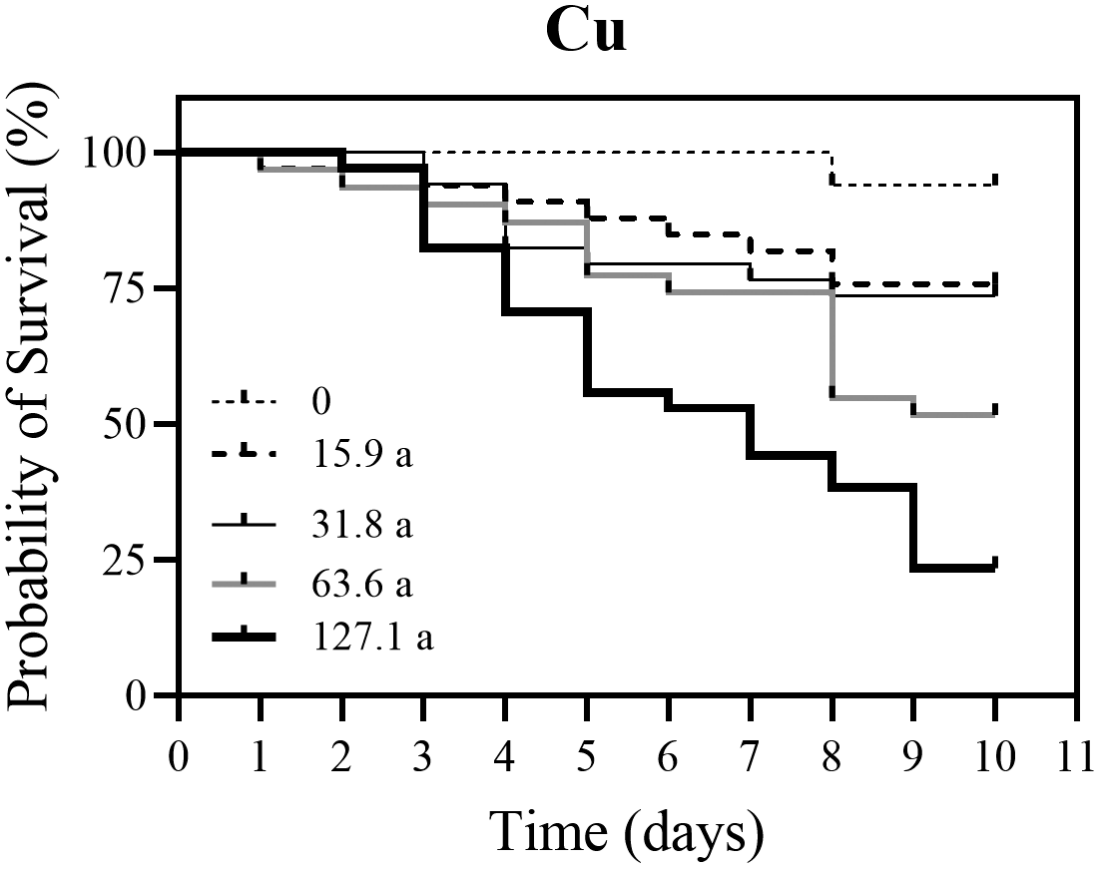
Kaplan–Meier survival curve of honey bee workers orally treated for 10 days with 15.9 mg/l (*N* = 32), 31.8 mg/l (*N* = 33), 63.6 mg/l (*N* = 33), and 127.1 mg/l (*N* = 34) Cu in 1.5 M sucrose as compared to the control (*N* = 33). Workers that were exposed to all concentrations of Cu showed significantly higher mortality than the control group. ^a^ Significant differences (Log-rank [Mantel-Cox] test): between control and 15.9 mg/l (χ^2^ = 4.26, *df* = 1, *P* = 0.0334), between control and 31.8 mg/l (χ^2^ = 5.395, *df* = 1, *P* = 0.0202), between control and 63.6 mg/l (χ^2^ = 15.30, *df* = 1, *P* < 0.0001) and between control and 127.1 mg/l (χ^2^ = 36.18, *df* = 1, *P* < 0.0001).

**Fig. 2.**
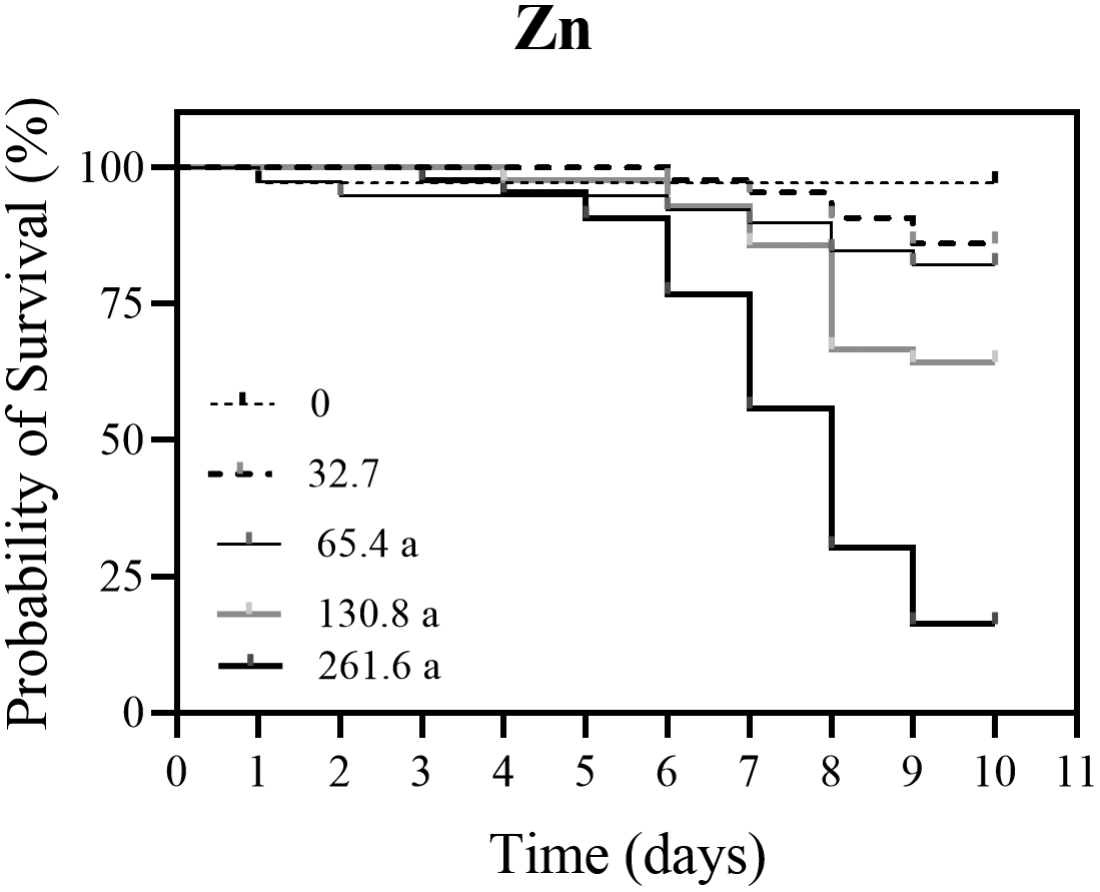
Kaplan–Meier survival curve of honey bee workers orally treated for 10 days with 32.7 mg/l (*N* = 42), 65.4 mg/l (*N* = 39), 130.8 mg/l (*N* = 42), and 261.6 mg/l (*N* = 44) of Zn in 1.5 M sucrose as compared to the control bees (0 mg/l Zn; *N* = 38). Workers that were exposed to concentrations 65.4, 130.8, and 261.6 of Zn showed significantly higher mortality than the control group. ^a^ Significantly different (Log-rank [Mantel-Cox] test): between control and 65.4 mg/l (χ^2^ = 4.171, *df* = 1, *P* = 0.0411), between control and 130.8 mg/l (χ^2^ = 11.75, *df* = 1, *P* = 0.0006), between control and 261.6 mg/l (χ^2^ = 47.82, *df* = 1, *P* < 0.0001).

For the oral chronic honey bee experiment, all Cu concentrations caused significantly higher mortality compared to the control group (0 mg/l Cu) (Log-rank (Mantel-Cox) test, χ^2^ = 45.84, *df* = 1, *P* < 0.0001, Fig. 1; Supplementary Table S1). Worker survival decreased with the increase in Cu concentration. A statistically significant difference was observed between the control group and bees treated with 15.9 mg/l Cu (Log-rank test, χ^2^ = 4.26, *df* = 1, *P* = 0.0334, Fig. 1), between the control group and bees treated with 31.8 mg/l Cu (Log-rank test, χ^2^ = 5.395, *df* = 1, *P* = 0.0202), between the control group and bees treated with 63.6 mg/l Cu (Log-rank test, χ^2^ = 15.30, *df* = 1, *P* < 0.0001) and between the control bees and bees treated with 127.1 mg/l Cu (Log-rank test, χ^2^ = 36.18, *df* = 1, *P* < 0.0001) (Fig. 1). For honey bee workers that were exposed to different concentrations of Zn, a significantly higher mortality was observed in groups treated with 65.4 mg/l Zn (Log-rank test, χ^2^ = 4.171, *df* = 1, *P* = 0.0411), 130.8 mg/l Zn (Log-rank test, χ^2^ = 11.75, *df* = 1, *P* = 0.0006), and 261.6 mg/l Zn (Log-rank test, χ^2^ = 47.82, *df* = 1, *P* < 0.0001) compared to the control group (0 mg/l Zn) (Fig. 2; Supplementary Table S2). Worker survival decreased with the increase in Zn concentration. No statistical difference in honey bee mortality was shown between the group treated with 32.7 mg/l Zn and control group (Log-rank test, χ^2^ = 2.751, *df* = 1, *P* = 0.0972). LC_50_ for Cu for 10-day exposure was estimated as 66.7 mg/l with 95% confidence intervals of 36.4 and 122.0 mg/l (slope [alpha] = 1.56). LC_50_ for Zn for oral 10-day exposure was estimated as 144.9 mg/l with 95% confidence intervals of 94.0 and 223.6 mg/l (slope [alpha] = 2.36).

**Fig. 3.**
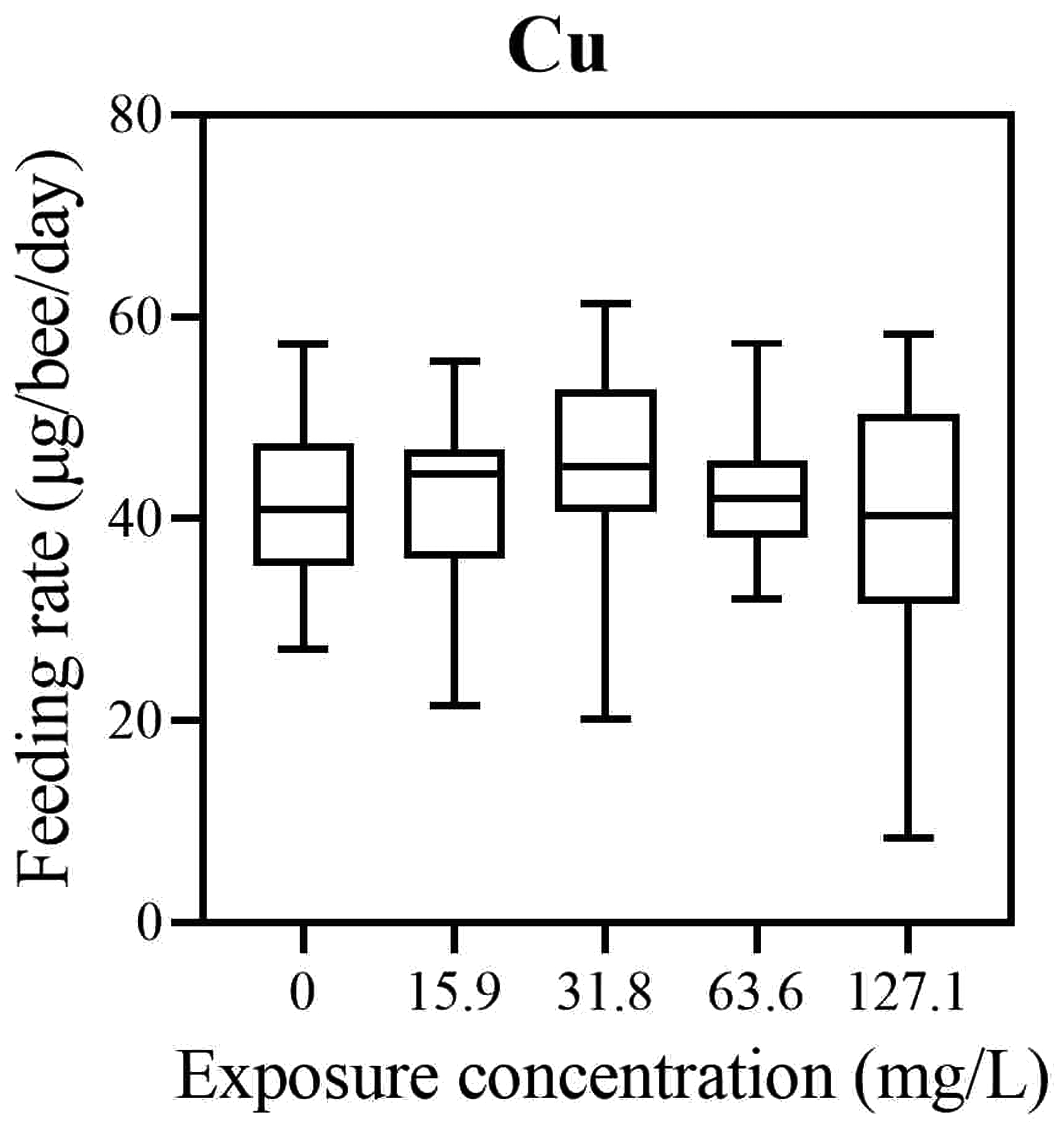
Feeding rate (amount of consumed food per day per honey bee) after chronic 10-day exposure to Cu with 15.9 mg/l (*N* = 32), 31.8 mg/l (*N* = 33), 63.6 mg/l (*N* = 33), and 127.1 mg/l (*N* = 34) Cu in 1.5 M sucrose as compared to the control (0 Cu mg/l) (*N* = 33). There was no significant difference in feeding rate patterns between experimental groups (Kruskal–Wallis, χ^2^ = 1.9076, *df* = 4, *P* = 0.7528).

**Fig. 4.**
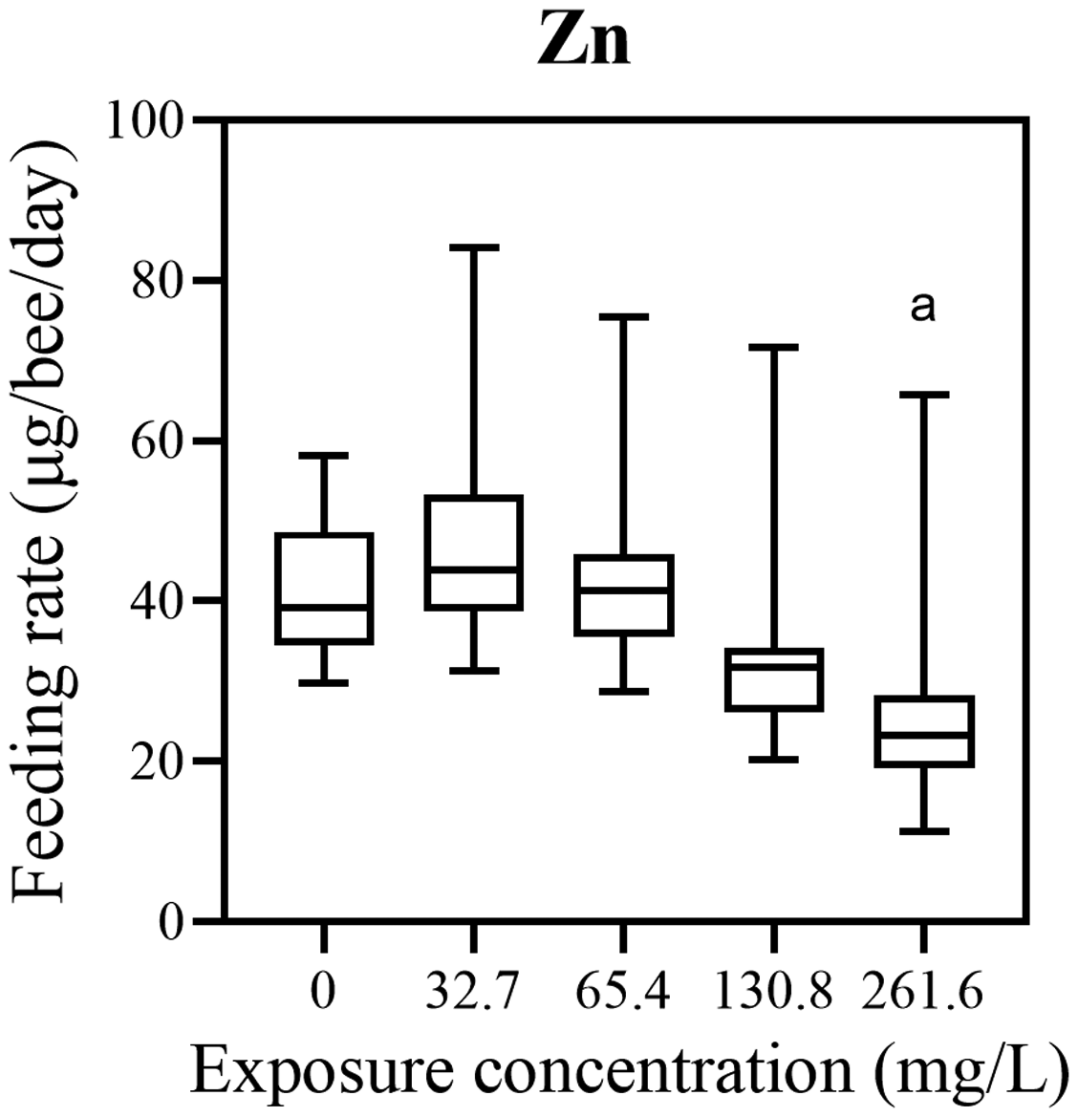
Feeding rate (amount of consumed food per day per honey bee) after chronic 10-day exposure to Zn with 32.7 mg/l (*N* = 42), 65.4 mg/l (*N* = 39), 130.8 mg/l (*N* = 42), and 261.6 mg/l (*N* = 44) of Zn in 1.5 M sucrose as compared to the control (0 mg/l of Zn; *N* = 38). There was a significant difference in feeding rate patterns between experimental groups (Kruskal–Wallis, χ^2^ = 20.651, *df* = 4, *P* = 0.0004). ^a^ Significantly different from control (Dunn’s post hoc test, *z* = 3.129, *P* = 0.0175), from 32.7 mg/l Zn-treated group (Dunn’s post hoc test, *z* = 3.804, *P* = 0.0014) and from 65.4 mg/l Zn-treated groups (Dunn’s post hoc test, *z* = 3.267, *P* = 0.0109).

No significant difference in feeding rate was determined between experimental groups treated with different concentrations of Cu (Kruskal–Wallis, χ^2^ = 1.9076, *df* = 4, *P* = 0.7528, Fig. 3; Supplementary Table S3). For the honey bees exposed to different concentrations of Zn, a significant difference in feeding rates between experimental groups was found (Kruskal–Wallis, χ^2^ = 20.651, *df* = 4, *P* = 0.0004, Fig. 4; Supplementary Table S4). The feeding rate of the 261.6 mg/l Zn-treated group was significantly different from the control (0 mg/l Zn) (Dunn’s post hoc test, *z* = 3.129, *P* = 0.0175), from 32.7 mg/l Zn-treated group (Dunn’s post hoc test, *z* = 3.804, *P* = 0.0014) and from 65.4 mg/l Zn-treated groups (Dunn’s post hoc test, *z* = 3.267, *P* = 0.0109). The feeding rate decreased with elevation of the concentration of Zn.

**Fig. 5.**
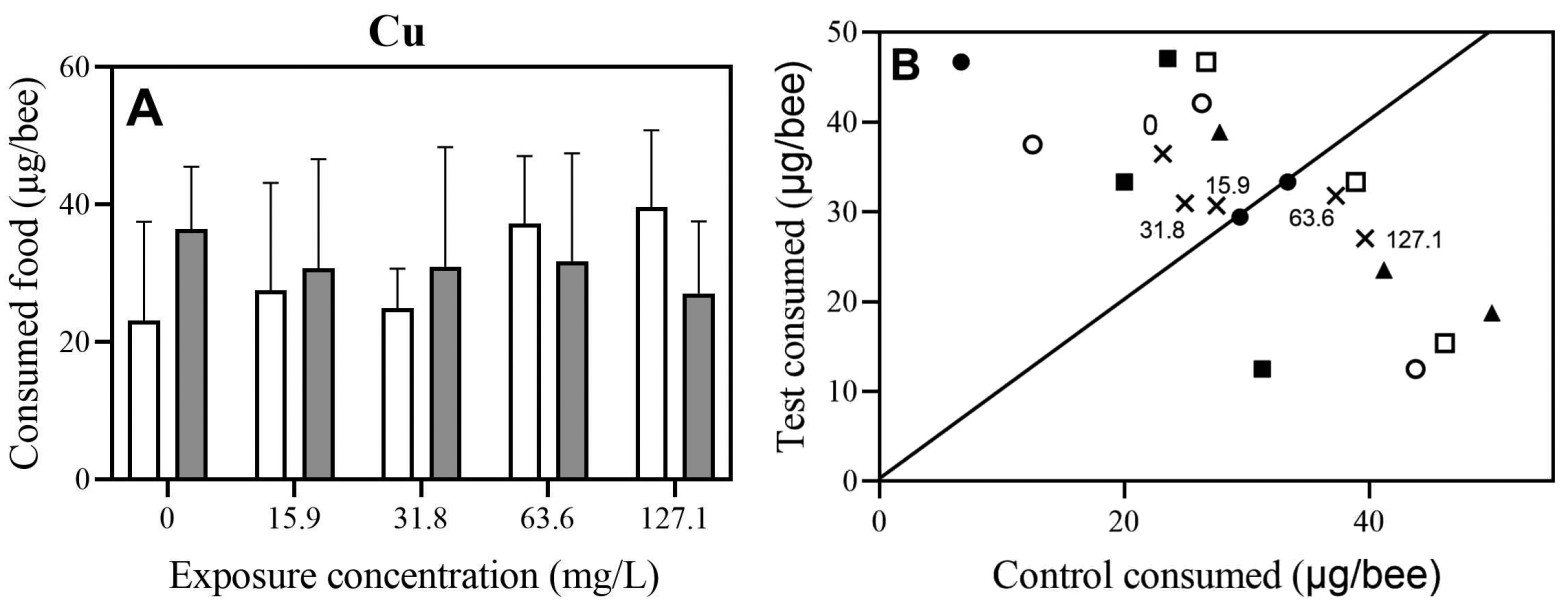
Effects of different concentrations of Cu on feeding in a 2-choice experiment after 24 h. A. Amount of consumed food in a 2-choice experiment expressed in µg/bee (control 1.5 M sucrose solution [empty bar] and 1.5 M sucrose suspensions of different Cu concentrations [dotted bar]). No statistically significant difference in feeding of honey bees was shown between consumed control 1.5 M sucrose solution and consumed test solutions (1.5 M sucrose solutions of different Cu concentrations) (Wilcoxon test, *V* = 45, *P* = 1, *P* = 0.666, *N* = 12). B. Relationship between consumed control 1.5 M sucrose solution (0 mg/l Cu) and consumed test solutions (1.5 M sucrose solutions of different Cu concentrations) in µg/bee. Cu concentrations were 0 mg/l (closed circles), 15.9 mg/l (open circles), 31.8 mg/l (closed squares), 63.6 mg/l (open squares), and 127.1 mg/l (closed triangles). Three test series for each test feeding solution were performed. The number of bees per concentration were: 0 mg/l (*N* = 50), 15.9 mg/l (*N* = 51), 31.8 mg/l (*N* = 48), 63.6 mg/l (*N* = 46), and 127.1 mg/l (*N* = 51). The solid line represents a 1-to-1 relationship. The concentrations used in the experiments are indicated in the graphs (A and B).

In a 24-h 2-choice feeding experiment where bees were offered 1.5 M sucrose solutions with different concentrations of Cu and a control 1.5 M sucrose solution without Cu, no statistically significant difference in feeding of honey bees was shown between the control 1.5 M sucrose solution and test solutions (1.5 M sucrose suspensions of different Cu concentrations) (Wilcoxon test, *V* = 45, *P* = 1) (Fig. 5A; Supplementary Table S5). The average ratios of the consumed Cu sucrose solutions containing 0, 15.9, 31.8, 63.6, or 127.1 mg/L Cu versus the consumed control solution were 1.58, 1.12, 1.24, 0.85, and 0.68, respectively (Fig. 5B, see the average values; Supplementary Table S5).

A progressive, but statistically not significant increase in the control 1.5 M sucrose and decrease in Cu consumption were observed with the increasing concentrations of Cu (Fig. 5A) showing that there is a tendency of deterrence in Cu for honey bees. The average consumption of the control sucrose solution increased from 23.14 µg/bee in a control group to 39.65 µg/bee in the group orally exposed to the highest concentration of Cu tested (127.1 mg/l Cu). The consumption of the test Cu solutions slightly decreased with elevated concentrations of Cu, ranging from 36.47 µg/bee in a control group down to 27.06 µg/bee when exposed to 127.1 mg/l Cu (Fig. 5A; Supplementary Table S5).

**Fig. 6.**
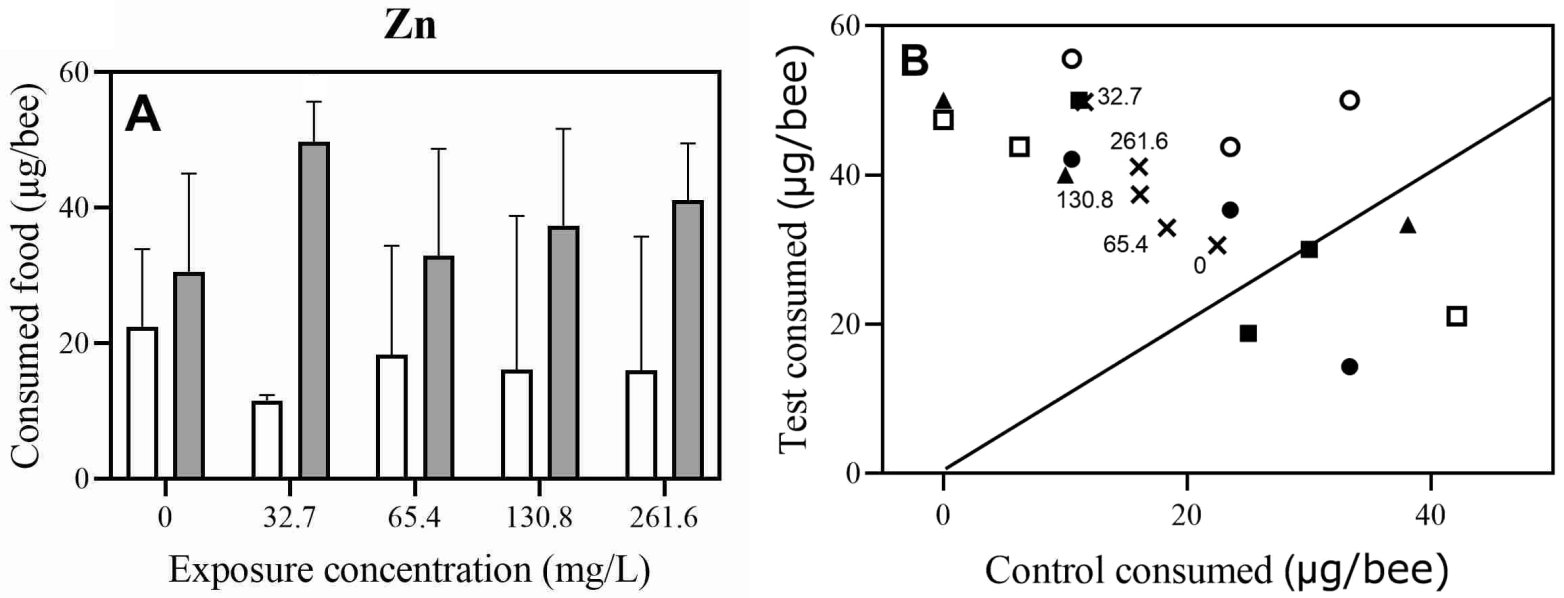
Effects of different concentrations of Zn on feeding in a 2-choice experiment after 24 h. A. Amount of consumed food in a 2-choice experiment expressed in µg/bee (control 1.5 M sucrose solution [empty bar] and 1.5 M sucrose suspensions of different Zn concentrations [dotted bar]). Statistically significant preference of honey bees for Zn over the control 1.5 M sucrose diet was shown by Wilcoxon test for paired samples, when comparing all tested concentration groups: 32.7, 65.4, 130.8, and 261.6 mg/l Zn (*V* = 12, *P* = 0.0120, *N* = 11). (B) Relationship between consumed control 1.5 M sucrose solution (0 mg/l Zn) and consumed test solutions (1.5 M sucrose suspensions of different Zn concentrations) in µg/bee. Zn concentrations were 0 mg/l (closed circles), 32.7 mg/l (open circles), 65.4 mg/l (closed squares), 130.8 mg/l (open squares), and 261.6 mg/l (closed triangles). Number of bees per concentration were: 0 mg/l (*N* = 57), 32.7 mg/l (*N* = 52), 65.4 mg/l (*N* = 54), 130.8 mg/l (*N* = 54), and 261.6 mg/l (*N* = 55). The solid line represents a 1-to-1 relationship. The concentrations used in the experiments are indicated in the graphs (A and B).

In a 24-h 2-choice feeding experiment where bees were offered 1.5 M sucrose solutions with different concentrations of Zn and a control 1.5 M sucrose solution without Zn, statistically significant preference of honey bees for Zn over the control 1.5 M sucrose diet was shown by Wilcoxon test when testing concentrations of 32.7, 65.4, 130.8, and 261.6 mg/l of Zn (*V* = 12, *P* = 0.0120) (Fig. 6A, see the average values; Supplementary Table S6). The highest preference for Zn was observed at the concentration of 32.7 mg/l Zn. The average ratios of consumed Zn sucrose solutions containing 32.7, 65.4, 130.8, and 261.6 mg/l of Zn versus the consumed control solutions were 1.36, 4.30, 1.80, 2.32, and 2.56, respectively (Fig. 6B, see the average values; Supplementary Table S6).

The food consumption in the 2-choice feeding experiments was also tested with the GLM procedure to discover relationships between observed variables. In the case of Cu, we were not able to find any model to observe a dependence of consumed food solutions on the concentration of Cu. Consumption of the test solution showed negative regression coefficient toward the consumed control solution (−0.74 ± 0.19, *R*^2^ = 0.54, *P* = 0.0018). The average consumed food showed some trend toward eating more of the control solution at higher concentration than test solution (Fig. 5B), but it was not statistically significant. Regarding Zn we found the contribution of concentration of Zn to the consumption rate of the test solution when compared with the control solution (Model construction for GLM: test ~ Zn concentration * control, Multiple *R*^2^ = 0.799, *F*_3,11_ = 14.53, *P* < 0.001). The concentration of Zn did not have significant effect on the consumption rate (GLM model, *P* = 0.11). On the contrary, the interaction of both variables (Zn concentration * control) had a significant effect on the consumption rate (GLM model, *P* = 0.031). The variability in the consumption rate of solutions of different Zn concentrations (between different experimental groups) was explained by the variability of the consumption rate of control solutions (GLM model, *P* < 0.001, negative coefficient = −1.19 ± 0.11). That means when the bees from the same group consumed more Zn-spiked solution, they consumed less of the control one. As seen from Fig. 6B, we can see that the biggest difference in consumption of the test solution toward the control was at the lowest concentration of Zn 32.7 mg/l. Also, at higher concentrations of Zn, the average consumption of the test solutions was higher than the control.

## Discussion

Our data from the survival curves of honey bee workers showed decreased survival with the elevation in concentration of Cu or Zn after 10-day oral exposure. The comparison of estimated LC_50_ values indicated that Cu has higher toxicity than Zn. As far as we know, no direct comparison between these metals within the same study on honey bees has been previously done. There are very limited studies of the influence of metals on honey bee survival in laboratory conditions. Di et al. (2016) conducted chronic and acute toxicity tests with Cd, Cu, and Pb contaminated food, but not with Zn. They found that Cu is least toxic to larvae and Pb to honey bee foragers. In the study of Di et al. (2016), the LC_50_ value for Cu for foragers was estimated as 72 mg/l, which is similar to our study, where the calculated LC_50_ value for Cu was 66 mg/l. However, these 2 estimated LC_50_ values are not directly comparable. Di et al. (2016) used Cu chloride dihydrate in 72-h oral exposure but tested only foragers. In our study, we evaluated the effects of Cu on in-hive honey bee workers, we used different cupric salts (CuSO_4_) and the duration period of exposure of bees was longer (10 days). Consequently, one could argue that in-hive honey bees are more resilient to Cu than foragers; however, more tests are needed to elucidate differential sensitivity to Cu among honey bee workers. In similar study with pesticides, foragers were consistently more susceptible to the flupyradifurone than in-hive bees (Tosi and Nieh 2019). Another study with the effects of Cu on bees was presented by Rodrigues et al. (2016). They evaluated the effects of oral exposure of neotropical stingless bees (*Friesella schrottkyi*) to high 24% Cu concentration and all bees died after 72 h, LT_50_ was estimated at 24 h. There is no relevant data about LC_50_ values for Zn salts in honey bees. In the study of Milivojević et al. (2015), the effects of chronic 10-day oral exposure of in-hive honey bee workers to 480 Zn mg/l were examined. The definitive survival rate of bees in Milivojević et al. (2015) after 10-day exposure was similar to the survival rates of bees in the present study; however, a noticeable drop in the survival rate was reported by Milivojević et al. (2015) after first 4 days of treatment.

Animal intoxication with pollutants can increase or decrease feeding rate (Mogren and Trumble 2010). Increased feeding could be the physiological stress response that is manifested as an increased energy mobilization and metabolic rate elevation needed for the activation of processes, such as detoxification, required to combat adverse effects of metals (Even et al. 2012). A decreased feeding in honey bees could be explained by the ability of bees to learn to avoid food cues after the preingestive and the postingestive negative consequences of toxins (Wright et al. 2010). Decreased feeding could also be due to sublethal effects that decrease metabolic activity like malaise effects. In this study, no difference in feeding rate was observed between different concentrations tested in bees chronically treated with Cu and control bees. In Di et al. (2016), foragers treated with metal consumed less of the supplemental 50% sucrose compared to controls, that was provided after feeding on metal solutions. We did not perform such test; we just monitored the feeding rate during consumption of Cu-spiked food.

We found that feeding rate decreased with the elevation of the concentration of Zn during the chronic oral experiment. Contrary to results presented here, Milivojević et al. (2015) showed elevated feeding rate in honey bees treated with 480 Zn mg/l as compared to the control, but almost double the concentration was used in the present study. This discrepancy could be attributed to different daily doses of Zn and thus different compensatory physiological mechanisms. The decrease in feeding was also observed in other Zn exposed invertebrates. Matthiessen et al. (1995) and Wilding and Maltby (2006) after Zn exposure demonstrated a reduced feeding rate in freshwater amphipod *Gammarus pulex*, while Drobne and Hopkin (1995) in terrestrial isopods.

To determine whether Cu or Zn are feeding deterrents or attractants for honey bees, 2-choice experiments were performed. We found there was a tendency for Cu to act as a deterrer for honey bees, however, it was not statistically significant. On the contrary, we showed that honey bees prefer Zn over the control 1.5 M sucrose diet. Behavioral deterrence is a mechanism used by animals to avoid the intoxication by harmful substances. Many studies have investigated the effects of metals on feeding preference or deterrence, especially for agricultural pests feeding on plants (Mogren and Trumble 2010). Cu, as copper sulfate (CuSO_4_), has been investigated as a feeding deterrent against agricultural pests for several lepidopteran species (El-Bassiouny 1991). They found out that different species were deterred at different concentrations of CuSO_4_. Zn salts, such as Zn sulfate, are also known feeding deterrents for lepidopteran agricultural pests (Mogren and Trumble 2010). Other invertebrates such as Locusts (*Schistocerca gregaria*), and slugs (*Deroceras caruanae*) avoid plants with high Zn content (Pollard and Baker 1997). A statistically significant preference for honey bees of Zn over the control 1.5 M sucrose diet was shown when statistically testing all concentrations of 32.7, 65.4, 130.8, and 261.6 mg/l of Zn. However, the highest preference for Zn was observed at the concentration of 32.7 mg/l Zn. The preference was shown also by our previous study, Glavan et al. (2017), for ZnO nanoparticles. It should be mentioned here that Zn ions dissolve from ZnO nanoparticles in the sucrose suspension (Milivojević et al. 2015) and physiological effects were induced by Zn ions, and not necessarily by nanoparticles.

Honey bees are at risk for those toxicants as they are unable to detect or recognize them as harmful. Using a 2-choice feeding experiment, it is possible to behaviorally evaluate the ability of animals to detect tested substances. Two mechanisms for detection of the effects of toxicants by honey bees were proposed: by gustatory receptors located on antennae/proboscis or by association of the sensory perception of the substance with the postingestive consequence of intoxication (Wright et al. 2010, de Brito Sanchez 2011). Possible gustatory perception of metals has not yet been described in honey bees. There is also a limited number of gustatory receptor genes and gustatory receptors found (de Brito Sanchez 2011). Burden et al. (2016) studied possible perception of selenium (Se) using the proboscis extension reflex conditioning and found out that Se is probably not detected through stimulation of gustatory receptors on the antennae or the proboscis as honey bees readily consume sucrose contaminated with lethal concentrations of Se (Burden et al. 2016). In another study by Burden et al. (2019), Cu was dose-dependently rejected by honey bees following antennal stimulation but was readily consumed following proboscis stimulation. The authors argued that mechanisms used for honey bee detection of Cu are probably present on antennae, but absent or less sensitive on the proboscis (Burden et al. 2019). In another study presented by Di et al. (2016), honey bee foragers when pretreated with high doses of Cu showed a decreased consumption of sucrose 24 h following ingestion of the metal. This decrease in the consumption was ascribed to malaise or postingestive consequence of intoxication by Cu (Burden et al. 2019). This is somewhat consistent with our result showing that there was a tendency in a deterrent property for Cu at high concentrations tested, so we can ascribe this tendency at some extent to a malaise condition in honey bees. No behavioral or physiological experiments on honey bees described above were performed with Zn, but our results show that honey bees exhibit preference for Zn over the control 1.5 M sucrose diet, indicating that they possess mechanisms to detect the presence of Zn in sucrose solution. It is also possible that honey bees are detecting something other than Zn in the solution, potentially Cl or the ion activity. Lau and Nieh (2016) demonstrated that honey bees show preference to water feeding stations with NaCl salt. Beneficial responses for Zn at low concentrations may also underlie the preference of Zn over the controls (Zhang et al. 2015, Nisbet et al. 2018).

Honey bees are exposed to metals predominantly by ingesting plant nectar and pollen. Hyperaccumulators may accumulate Cu and Zn. However, Zn is much more prone to accumulate than Cu (Lange et al. 2017). Approximately 28 plant species have been described as Zn hyperaccumulators, but the levels of Zn accumulation vary between the species and between different parts of the plants (Balafrej et al. 2020). The use of hyperaccumulator plants for metal remediation in soil, which are frequently visited by honey bees for nectar or pollen, could pose a potential danger for bees. However, there is not much data about the concentrations of Zn and Cu in nectar and pollen, and the concentrations of metals vary in different plant species (Di et al. 2020). In the pollen, the levels of Cu, Mn, Pb, and Zn vary from 5.44 to 11.75 μg/ml; 34.31 to 85.75 μg/ml; 13.98 to 18.19 μg/ml; and 50.19 to 90.35 μg/ml, respectively (Silva et al. 2012). Thus, it is difficult to evaluate the risk of honey bee exposure to realistic concentrations of Zn and Cu in their main food source. In this study, the concentrations of Cu were 15.9–127.1 mg/l and of Zn 32.7–261.6 mg/l, and are comparable with measured or estimated environmental concentrations. The soil of polluted areas in the United States contained 41.17 mg/kg of Cu, Cu concentrations ranged from 0.06 to 495 mg/kg (Holmgren et al. 1993, Meng et al. 2016). The measured concentrations of Zn in different areas were 0.002–50 mg/l in water (Warren 1981), <5–2,000 mg/kg in soil (Schacklette and Boerngen 1984) and <1 mg/m^3^ in air (Lloyd and Showak 1984).

Cu and Zn may also accumulate in bees and bee products. In honey bees, the range of Cu concentrations from urban, industrialized, or agricultural-woodland regions varied between 12 and 37 mg/kg, whereas the concentrations measured for Zn were higher, between 45 and 76 mg/kg (Leita et al. 1996, Fakhimzadeh and Lodenius 2000, Roman et al. 2010, Giglio et al. 2017). In honey and propolis the measured concentrations were lower: in honey 0.82–0.18 mg/kg of Cu and 3.58 mg/kg of Zn and in propolis 18.1 mg/kg of Cu and 6.95 mg/kg of Zn (Roman et al. 2010, 2011). Potential future studies could include testing bioaccumulation of metals in bees, especially queens, who have longer lifespans. Additionally, recommendations to farmers can be made, such as using alternative methods of introducing micronutrients to plants, like modifying soils to increase bioavailability of metals rather than applying micronutrients.

Our study delivers baseline information about the chronic oral effects of Cu and Zn on the survival and feeding rate of in-hive honey bee workers. However, to fully understand the toxicity of these 2 metals in honey bees other casts and foragers must also be tested. One of the shortcomings of study was the use of in-hive honey bee workers of different ages and 3 replicates per each test that probably contributes to the variability of the results. This kind of laboratory test examines toxicity under worst-case exposure conditions. To provide realistic conditions, semifield, and field studies on honey bee colonies must be performed (EPO 2010).

In conclusion, we found out that Cu and Zn induce mortality at concentrations comparable with measured concentrations in the environment. The calculated LC_50_ values are within reasonable environmental concentrations for these metals. The current study also shows that honey bees prefer sucrose solution when contaminated with Zn, but not with Cu, indicating that they are probably able to detect Zn in sucrose-spiked solutions. Cu is more toxic for honey bees than Zn, but the preference for Zn might elevate the possible risk for intoxication in a Zn-polluted environment. Finally, our results bring the alert to the mindful use of Zn in soil for agriculture since this metal is a vital micronutrient for plants and there are many strategies proposed for enhancing Zn plant availability. Cu is not easily accumulating in plants, so a precautious use of CuSO_4_ as a fungicide near the bee hives is recommended.

## Supplementary Material

Supplementary material is available at Journal of Economic Entomology online.

toae108_suppl_Supplementary_Table_S1

toae108_suppl_Supplementary_Table_S2

toae108_suppl_Supplementary_Table_S3

toae108_suppl_Supplementary_Table_S4

toae108_suppl_Supplementary_Table_S5

toae108_suppl_Supplementary_Table_S6
